# Finnish new variant of *Chlamydia trachomatis* escaping detection in the Aptima Combo 2 assay also present in Örebro County, Sweden, May 2019

**DOI:** 10.2807/1560-7917.ES.2019.24.26.1900370

**Published:** 2019-06-27

**Authors:** Magnus Unemo, Marit Hansen, Ronza Hadad, Ylva Lindroth, Hans Fredlund, Mirja Puolakkainen, Martin Sundqvist

**Affiliations:** 1 World Health Organization Collaborating Centre for Gonorrhoea and Other Sexually Transmitted Infections (STIs), National Reference Laboratory for STIs, Department of Laboratory Medicine, Faculty of Medicine and Health, Örebro University, Örebro, Sweden; 2 Department of Laboratory Medicine, Medical Microbiology, Lund University, Skåne Laboratory Medicine, Lund, Sweden; 3 Department of Virology and Immunology, University of Helsinki and Helsinki University Hospital, HUSLAB, Helsinki, Finland

**Keywords:** Chlamydia trachomatis, NAAT, nvCT, Finland, Sweden, Diagnostics

## Abstract

We identified the first two cases of the Finnish new variant of *Chlamydia trachomatis* (F-nvCT) beyond Finland in two clinical urogenital specimens in Örebro County, Sweden. These Aptima Combo 2 assay-negative specimens were Aptima *Chlamydia trachomatis* (CT) assay positive and had the characteristic C1515T mutation in the 23S rRNA gene. From 22 March to 31 May 2019, 1.3% (2/158) of the CT-positive cases in Örebro County were missed because of the F-nvCT. International awareness, investigations and actions are essential.

Recently, false-negative *Chlamydia trachomatis* (CT) specimens in the nucleic acid amplification test (NAAT) Aptima Combo 2 (AC2) (Hologic Inc., San Diego, California, United States (US)) detecting CT (target: 23S rRNA) and *Neisseria gonorrhoeae* (target: 16S rRNA) were reported in Finland [1]. AC2 CT-negative/equivocal specimens, mostly having relative light unit (RLU) signals of 20–85, were confirmed as CT positive using the Aptima *Chlamydia trachomatis* assay (ACT) (target: 16S rRNA) [1]. A C1515T mutation in the CT 23S rRNA gene was confirmed as the reason that the Finnish new variant of CT, F-nvCT, escapes detection in AC2 [2]. It is essential to timely investigate presence of F-nvCT internationally. Finland and Sweden have close relationships, including geographic proximity, and travelling between the two countries is frequent.

We examined: (i) the proportion of consecutive clinical AC2 CT-negative/equivocal specimens (RLUs 20–99; *N. gonorrhoeae* negative) from 1 January 2017 to 31 May 2019; (ii) AC2 CT specimens (negative, equivocal and positive) and their RLUs from 1 May 2018 to 31 May 2019; (iii) results from ACT testing of AC2 CT-negative/equivocal specimens (RLUs 20–99; *N. gonorrhoeae *negative) from 22 March 2019 to 31 May 2019 in Örebro County, Sweden; and (iv) partial 23S rRNA gene by sequencing (personal communication, K Hokynar, May 2019) in AC2 CT-negative/equivocal specimens that were ACT positive.

## *Chlamydia trachomatis* diagnostics and incidence

**Figure 1:**
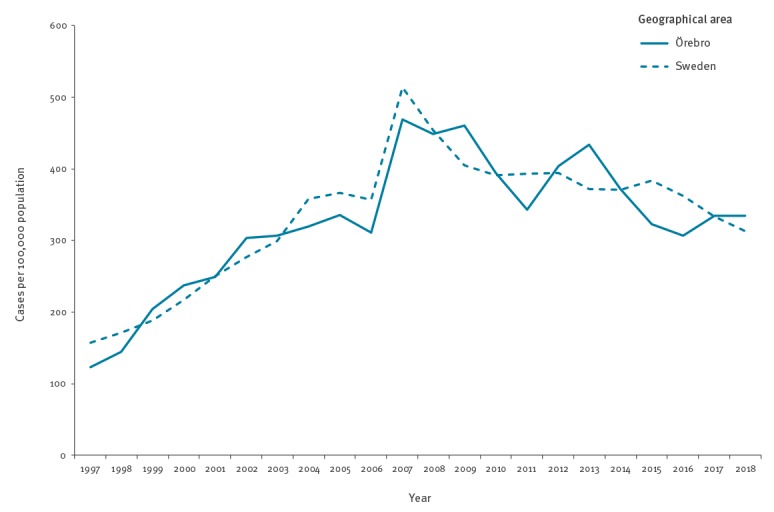
Incidence of mandatorily reported *Chlamydia trachomatis* infection in Örebro County, Sweden and national Swedish incidence, 1997–2018

In Örebro County (ca 300,000 inhabitants), Sweden, all CT and *N. gonorrhoeae* samples are analysed at the Örebro University Hospital using AC2 on a Panther instrument (Hologic Inc., San Diego, US). The incidence per 100,000 inhabitants of mandatorily reported CT cases in Örebro County has been similar to the national incidence and has decreased from 2013 (434/100,000 population) to 2018 (334/100,000 population) (Figure 1).

An increased incidence was seen in 2007 because of the introduction of new nucleic acid amplification tests (NAATs) that also detected the Swedish new variant of *Chlamydia trachomatis* (nvCT). It was a ‘catch up’ of samples missed because of the Swedish nvCT.

## Initial investigations of possible Aptima Combo 2 false-negative *Chlamydia trachomatis* specimens

All consecutive clinical AC2 specimens obtained at Örebro University Hospital, Sweden from 1 January 2017 to 31 May 2019 (n = 49,189, mainly vaginal swabs and urine specimens) were assessed.

Based on the initial data from Finland, nearly all false-negative AC2 CT specimens had RLUs of 20–85. Consequently, we decided to evaluate specimens with these AC2 RLUs in Örebro County, Sweden and additionally AC2 RLUs of 86–99 to cover all AC2 CT equivocal specimens, i.e. RLUs 25–99. The proportion of AC2 specimens with RLUs 20–99 was low from January 2017 to September 2018, ranging between 0.07% and 0.71%, but from October 2018 onwards, the proportion was substantially higher (range: 1.26–2.94%) (Table 1). This prompted further investigations.

**Table 1:** Number and proportion of Aptima Combo 2 *Chlamydia trachomatis* (CT)-negative/equivocal specimens with relative light unit signals of 20–99 of all Aptima Combo 2 CT-negative specimens, Örebro County, Sweden, January 2017–May 2019

Month	2017		2018		2019	
	n/N	%	n/N	%	n/N	%
January	3/1,672	0.18	2/1,793	0.11	47/1,947	2.41
February	4/1,682	0.24	6/1,572	0.38	18/1,434	1.26
March	6/1,815	0.33	6/1,575	0.38	22/1,598	1.38
April	8/1,496	0.53	7/1,652	0.42	33/1,316	2.51
May	5/1,581	0.32	1/1,516	0.07	34/1,599	2.13
June	11/1,554	0.71	3/1,222	0.25	NA	NA
July	6/1,356	0.44	5/1,482	0.34	NA	NA
August	4/1,772	0.23	5/1,722	0.29	NA	NA
September	3/1,726	0.17	6/1,727	0.35	NA	NA
October	4/1,726	0.23	23/1,741	1.32	NA	NA
November	9/1,775	0.51	29/1,513	1.92	NA	NA
December	9/1,403	0.64	39/1,328	2.94	NA	NA

NA: not applicable.

**Figure 2:**
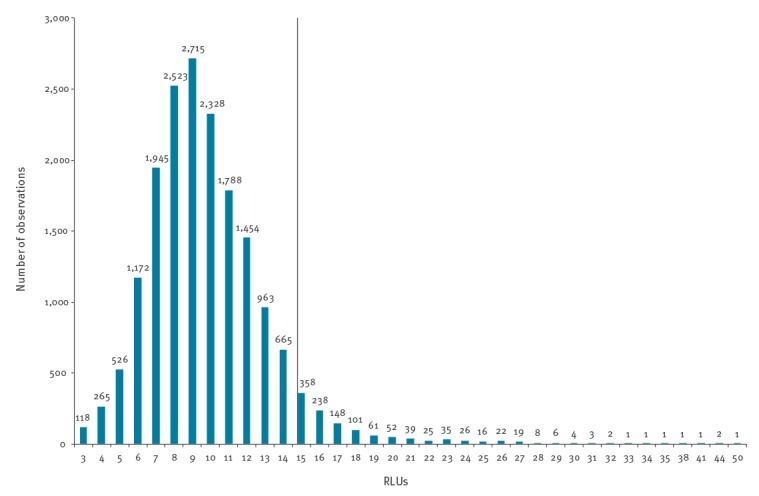
Relative light units of *Chlamydia trachomatis* and *Neisseria gonorrhoeae*-negative Aptima Combo 2 specimens, Örebro County, Sweden, May 2018–May 2019 (n = 17,633)

Of 19,083 AC2 results from 1 May 2018 to 31 May 2019, 1,205 (6.3%) were reported as CT positive, 17,865 (93.6%) as CT negative and 13 (0.07%) as CT equivocal (RLU range for these specimens: 32–98). The RLUs of the AC2 CT and *N. gonorrhoeae-*negative specimens (n = 17,633) are shown in Figure 2. Briefly, the mean RLU was 9.8, 75% of observations were RLUs ≤ 11, 90% were RLUs ≤ 14, 95% were RLUs ≤ 15 and 99% RLUs were ≤ 21 (Figure 2).

RLUs: relative light units.

The black line (RLU ≥ 15) shows the currently recommended cut-off for confirmatory reflex testing using the Aptima *Chlamydia trachomatis* assay [2].

The mean RLU was 9.8, while the median was 9 and the range 3–50. Furthermore, 75% of observations were RLUs ≤ 11; 90% were ≤ 14; 95% were ≤ 15; 96% were ≤ 16; 97% were ≤ 17; 98% were ≤ 18; and 99% were ≤ 21.

## Identification of possible cases of the Finnish new variant of *Chlamydia trachomatis*

Promptly after the first indication in March 2019 of possibly false-negative AC2 CT specimens, mostly having RLUs of 20–85, in Finland, confirmatory reflex testing using ACT of all specimens with AC2 RLUs 20–99 was implemented at Örebro University Hospital. Briefly, from 22 March to 31 May 2019, 156 specimens were AC2 CT positive and 3,485 were CT negative. There were 77 negative/equivocal specimens with RLUs 20–99 (RLUs 20–24: n = 53, RLUs 25–30: n = 19, and RLUs 31–78: n = 5) and *N. gonorrhoeae-*negative results. Seventy-five (97.4%) of these specimens were interpreted as CT negative and two (2.6%) as CT equivocal by the Panther instrument. Seventy (90.9%) of the 77 specimens were available for ACT testing. One AC2 CT-equivocal specimen (RLU 78) and two AC2 CT-negative specimens (RLUs 30 and 32) were ACT positive (RLUs 4,318–7,168). However, the AC2 CT-equivocal specimen was also positive in repeated AC2 testing (RLU 729).

## Confirmed cases of the Finnish new variant of *Chlamydia trachomatis*

Both the two repeatedly AC2 CT-negative/ACT-positive specimens contained the CT 23S rRNA C1515T mutation, which was recently confirmed as the cause of the false-negative/equivocal AC2 CT results of F-nvCT specimens [2]. These two Swedish F-nvCT specimens were sampled in May 2019 from one Swedish male and one Swedish female in their early 20s. Both patients received CT-positive results based on the ACT testing, and recommended treatment [3]. The female had one unknown sexual contact in Norway. The male named several female sexual contacts in Sweden, one of whom was from Finland who however tested negative for CT in late May 2019.

## Discussion

We identified the first two cases of F-nvCT beyond Finland, in two clinical specimens in Örebro County, Sweden. Correspondingly, from 22 March to 31 May 2019, 1.3% (2/158) of the CT-positive cases in Örebro County were missed because of the F-nvCT. Further investigations are ongoing in Örebro County and in the two additional Swedish laboratories using AC2. One of these laboratories examined more than 110,000 AC2 specimens in 2018, and the proportion of specimens with AC2 RLUs of 20–99 has been stably low since 2016 (0.15–0.16%). Detailed examination has now been initiated in this laboratory as well. Based on the close relationships between Finland and Sweden, it is likely that additional F-nvCT cases will be detected in Sweden. It is highly important to perform a timely F-nvCT surveillance study in the Swedish capital city, Stockholm. Stockholm is geographically close to Finland and travelling between Stockholm and many Finnish cities with airplanes and ferries is frequent.

It is essential to detail the national proportion and geographic distribution of cases of F-nvCT in Finland and Sweden, and possible presence in additional countries. Hologic, the manufacturer of the AC2, is developing an Aptima-format research assay containing an F-nvCT detection probe for surveillance, particularly in European settings where elevated levels of AC2 CT false-negative/equivocal results have been verified [2]. When such an assay is developed and validated, a well-designed, pan-European, manufacturer-independent F-nvCT surveillance study would be valuable. Notably, only the CT probe detection in AC2, and not the AC2 target capture or transcription-mediated amplification, is affected by the F-nvCT. Furthermore, other commercially-available US Food and Drug Administration-approved CT NAATs detect the F-nvCT.

At current date, European laboratories using AC2 should retrospectively and prospectively review their CT results, the proportions of negative, equivocal, and positive specimens, and the RLUs of all negative/equivocal specimens, and examine unexplained changes in the CT epidemiology or positivity rate. In European settings, until a revised version of AC2 that detects also F-nvCT is available, specimens with AC2 RLUs 15–99, CT-negative specimens that are *N. gonorrhoeae *equivocal/positive and CT-equivocal specimens independent on *N. gonorrhoeae* results should be reflex tested with ACT [2,4]. This will identify possible cases of F-nvCT specimens. To identify confirmed cases of F-nvCT specimens, CT 23S rRNA gene sequencing of specimens that are AC2 CT negative/equivocal and ACT positive is essential until other mutant-specific assays are available. Where false-negative AC2 CT tests have been reported to patients, patient recall policies need to be implemented. The look-back period will depend on the local epidemiology of F-nvCT, taking spontaneous clearance of CT infection, social consequences and potential risk of reinfection into account.

In due course, many scientific issues should be elucidated, such as where and when and how the F-nvCT emerged and is spreading, how it is evolving and the fitness of F-nvCT nationally and internationally. Moreover, it should be studied if the F-nvCT is associated with increases in CT-associated complications/sequelae, symptomatic/asymptomatic infection and spread in different subpopulations. Whole genome sequencing of F-nvCT from Aptima specimens is in progress to address several scientific issues. A mutant-specific, real-time PCR is also under development. Notably, the CT 23S rRNA C1515T mutation in F-nvCT has not been found in previously published CT genome sequences [5] and is most likely not associated with any resistance to first- or second-line therapy with azithromycin [3].

In general, for CT infections and other infections, it is imperative to closely monitor and analyse incidence, locally, nationally and internationally. It is also important to alert and further examine unexplained notable decreases or increases in diagnosis rates in a timely manner. Furthermore, regular and more comprehensive evaluations of different diagnostic methods are crucial for maintaining diagnostic quality. The samples included in such evaluations should reflect not only currently circulating strains, but also temporally, geographically and genetically diverse strains. Frequent participation is also crucial in appropriate external quality assessments schemes, which should ideally include similar diverse strains, different diagnostic methods and divergent populations. Based on the identification of the Swedish nvCT in 2006 [6-9], *N. gonorrhoeae* *porA* pseudogene mutants [10,11] and the F-nvCT [1,2], diagnostic test escape mutants of CT and other infectious agents may be more common than realised and inevitable consequences of the frequent use of NAATs and the ongoing evolution of the microorganisms. Accordingly, two targets might need to be considered in all diagnostic NAATs [7,9,10]. International and national surveillance programmes capturing diagnostic test escape mutants, and cross-reacting microorganisms, for CT and other pathogens should also be considered, particularly for diagnostic NAATs with a single target. Finally, preservation and/or confirmatory testing of 1–5% of representative negative/equivocal NAAT specimens each year would be valuable.
